# Case report of central serous chorioretinopathy with intraretinal fluid and normal fundus fluorescein and indocyanine green angiography

**DOI:** 10.1093/jscr/rjae723

**Published:** 2024-11-26

**Authors:** Manasi Hegde, Travers Weaver

**Affiliations:** Department of Opthamology, Princess Alexandra Hospital, Queensland Health, 99 Ipswich Rd, Woolloongabba 4012, Australia; Department of Opthamology, Princess Alexandra Hospital, Queensland Health, 99 Ipswich Rd, Woolloongabba 4012, Australia

## Abstract

A 73-year-old man was diagnosed with central serous chorioretinopathy (CSCR). He had atypical features including a normal indocyanine green angiography (ICG) and fundus fluorescein angiography (FFA), uncommon age group for initial diagnosis and a finding of intraretinal fluid. This case report is the first of our knowledge that exemplifies this type of unusual clinical presentation for CSCR.

## Introduction

Central serous chorioretinopathy (CSCR) is the fourth most common retinal disease with 9.9 of 100 000 men and 1.7 of 100 000 women affected [1]. Despite its high incidence, the pathophysiology isn’t well understood. A pathophysiology proposed is that the hyperpermeability of choroid capillaries results in retinal pigment epithelial (RPE) dysfunction [2]. CSCR is correlated with prior steroid use, *Helicobacter pylori* infection, Type A personality, pregnancy, and hyperopia [3].

The most common presenting symptoms are central scotoma, reduced contrast sensitivity, micropsia, reduced colour saturation, refraction shift, metamorphopsia [1].

The natural course of acute CSCR is self-limiting within 2–3 months [1]. Chronic CSCR is defined as persistent subretinal fluid for more than 6 months [4].

We present an atypical case of unilateral chronic CSCR. To our knowledge, there is no previous literature demonstrating this.

## Case report

A 73-year-old male self-presented to the eye emergency with 2 weeks of left blurred vision and loss of contrast. The onset of symptoms were a few days following chemotherapy for diffuse, large B cell lymphoma. He had a known history of poor vision in his right eye since childhood. Otherwise, his background included hypertension.

**Figure 1:**
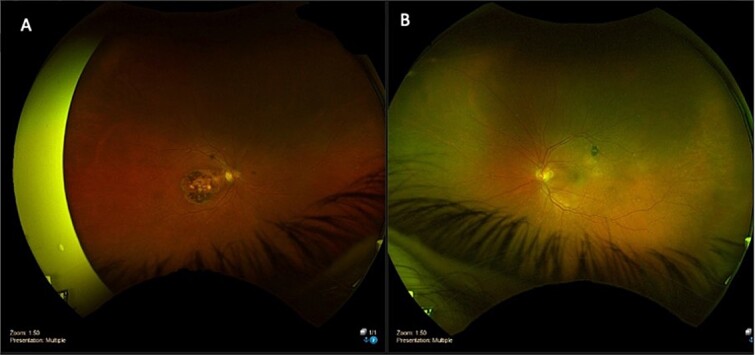
Optomap fundus photos of the patient. (A) Right eye with macular atrophy. (B) Left eye with macular subretinal fluid (SRF) and retinal pigment epithelial (RPE) detachment at the superior arcade.

On examination, his corrected visual acuity on Snellen chart was 6/9.6-2 for his left eye and 6/60 for his right eye (baseline). Anterior segment examination was unremarkable. On dilated fundus exam, his right eye had longstanding macular atrophy. His left eye had a 0.3 pink disc, no swelling, with a blunted macular reflex. This can be seen in Fig. 1.

**Figure 2:**
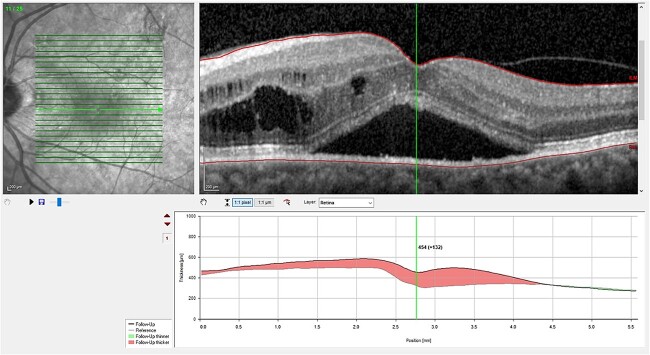
(A) OCT of left eye – IRF and SRF with serous retinal detachment. Choroidal thickness = 454 μm.

**Figure 3:**
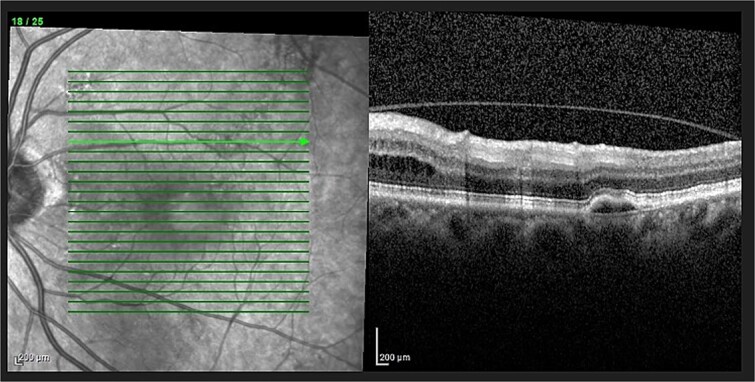
OCT of left eye – showing IRF and pigment epithelial detachment.

Optical coherence tomography (OCT) demonstrated an atrophied right macular. Whilst the left eye showed a thickened choroid, multiple pigment epithelial detachments (PED), subretinal fluid and intraretinal fluid nasally. This is shown in Figs 2 and 3.

**Figure 4:**
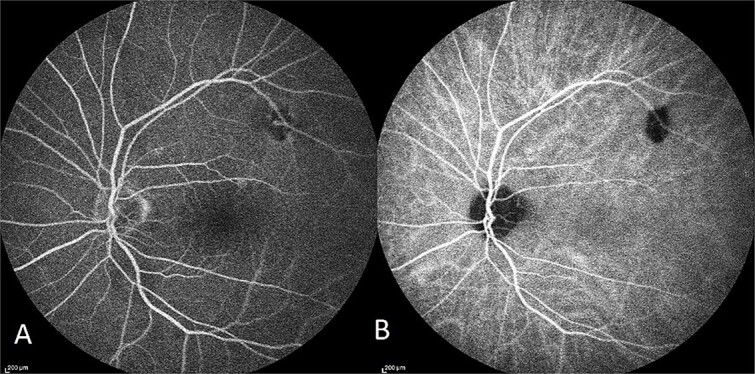
Capture of the patient’s left eye FFA (A) and ICG (B) in midphase. Hypofluorescence along the superior arcade is an area of incidental poor choroidal perfusion.

The patient had normal macular FFA and ICG as seen in Fig. 4.

The patient was diagnosed with CSCR.

In the first instance, it was presumed that the CSCR was secondary to prednisone from his chemotherapy regime. He had no family history of CSCR, no symptoms of *H. pylori* infection and no underlying refractory error. In discussion with his haematologist, the steroid therapy was weaned and ceased.

Despite 9 months since cessation of steroid therapy, the patient had persistent left subretinal and intraretinal fluid and fluctuating corrected visual acuity ranging from 6/6-2 to 6/9. His ophthalmologist opted against invasive treatment as it was the patient’s better seeing eye. This was now called chronic CSCR, and the decision was made to monitor.

**Figure 5:**
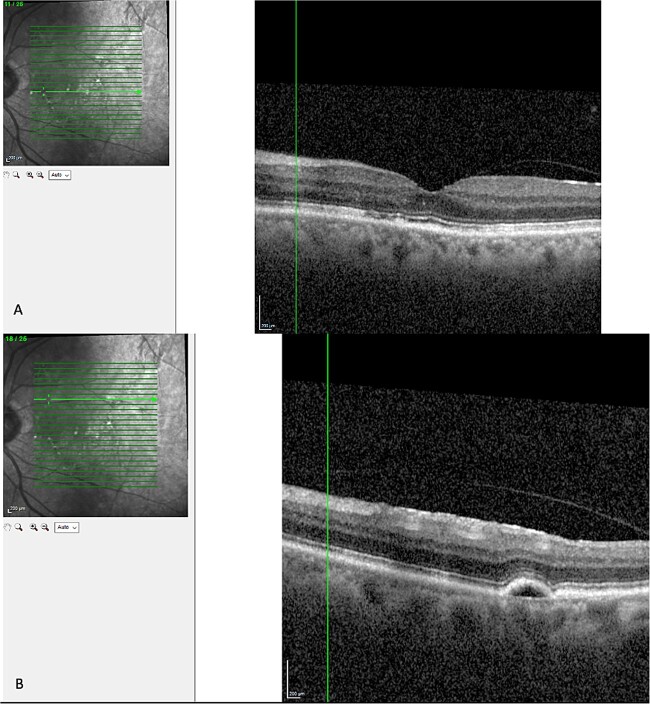
Latest OCT of the patient’s macular. (A) Shows absence of intraretinal or subretinal fluid. (B) Shows a pigmental epithelial detachment.

Finally, the patient was seen 12 months later. His left eye vision was 6/9 and his OCT showed a serous PED, with resolution of subretinal or intraretinal fluid as seen in Fig. 5.

## Discussion

The diagnosis of CSCR was made by the patient’s ophthalmologist, meeting diagnostic criteria as seen in Table 1. The patient meets major criteria with an OCT showing serous retinal detachment, and RPE alterations seen in Figs 2 and 3, respectively. Figure 2 displays a subfoveal choroidal thickness of 454 μm meeting the minor criteria.

**Table 1:** Clinical diagnostic criteria for central serous chorioretinopathy [5]

Major criteria – must have both of the following:	Minor criteria – must have at least 1 of the following:
Presence of serous retinal detachment on OCT One area of RPE alteration on fundus autofluorescence, OCT or infrared imaging	Mid-phase hyperfluorescent placoid areas on ICG One or more focal leaks on FFA Subfoveal choroidal thickness of 400 μm or more

Importantly, this patient had clinical features inconsistent with intraocular lymphoma with no anterior or posterior segment inflammation, no optic nerve swelling and no retinal or subretinal lesions across multiple visits in 2 years [6]. Intracerebral disease was excluded on magnetic resonance brain imaging. Hypergammaglobinaemia disorders were considered due to the normal FFA and ICG and the history of B cell lymphoma. However, the patient had normal serum immunoglobulins. Additionally, a bone marrow aspirate demonstrated normal plasma cells and morphology.

There are a few aspects of his case that makes it an atypical chronic CSCR.

Firstly, he was outside of the mean age of incidence for CSCR which was found to be 41 and 39 years of age in two cohort studies, respectively [7, 8]. This exemplifies the importance of still considering CSCR in older age groups.

Secondly, CSCR usually has supporting features for the diagnosis on ICG and FFA which are not demonstrated in this case. The most common signs on FFA at the macular are a pinpoint leak in the early phase, at the mid-phase, the hyper-fluorescence increases in intensity and in the late-phase evolves into a ‘smokestack’ or ‘ink-blot’ pattern [9]. ICG, in CSCR, displays choroidal filing delay in the early phase, hyperpermeability of choroidal vessels with multi-focal hyper-fluorescence around the macular in the mid-phase and fading of this hyper-fluorescence in the late phase [9, 10]. As demonstrated in Fig. 4, the patient had a normal FFA and ICG in mid-phase.

Finally, the patient had intraretinal fluid. A study found that hyper-reflectivity in the outer nuclear layers was described in over 90% of CSCR patients and this correlated with subretinal fluid. Intraretinal fluid wasn’t seen [11]. This demonstrates the importance of still considering CSCR in patients with intraretinal fluid.

Treatment for CSCR doesn’t have a consensus amongst clinicians. Management includes laser photocoagulation, intravitreal anti-vascular endothelial growth factor injections, and mineralocorticoid or glucocorticoid receptor antagonist [12]. Treatment options are limited for this individual. Firstly, steroid receptor antagonist therapy cannot be implemented given the patient’s co-existing medical condition of diffuse large B cell lymphoma. Additionally, laser therapy or intravitreal injections were considered; however, the treating ophthalmologist believed it was high risk due to the contralateral eye having longstanding poor vision.

Although the patient’s subretinal and intraretinal fluid has resolved; It’s possible that he will have a recurrence of CSCR as a previous study found that 31% of patients had recurrence [7].

## Conclusion

The key points from this case are to consider CSCR as a differential in older patients, those with a normal ICG or FFA and those with intraretinal fluid. It’s important to consider CSCR for patients presenting similarly as it can be self-resolving and not require invasive treatment.

## Data Availability

The data that support the findings of this study are included in the published manuscript.
